# Dissociative Identity Disorder Cotreated With Zinc and L-carnosine: A Case Report

**DOI:** 10.7759/cureus.74794

**Published:** 2024-11-29

**Authors:** Kensaku Sakae, Machi Suka, Hiroyuki Yanagisawa

**Affiliations:** 1 Department of Psychiatry, Keieikai Yashio Hospital, Saitama, JPN; 2 Department of Public Health and Environmental Medicine, The Jikei University School of Medicine, Tokyo, JPN

**Keywords:** binge eating, bipolar disorder, dissociation, dissociative identity disorder, flashback, glutamate, l-carnosine, self-harm, zinc, zinc deficiency

## Abstract

Little is known about the effectiveness of pharmacotherapy in dissociative identity disorder (DID). Zinc is essential for proper brain function. Its deficiency can lead to mental health symptoms, possibly contributing to dissociation. L-carnosine is an endogenous dipeptide with a neuroprotective effect. We report on the case of a 30-year-old woman with DID and comorbid bipolar I disorder who had zinc deficiency and was successfully cotreated with zinc and L-carnosine. She displayed three alternate identities and exhibited signs of emotional/mood instability, flashbacks, binge eating, and self-harm. The patient also displayed several physical symptoms of zinc deficiency. She did not respond to aripiprazole (0.75 mg/d) and clonazepam (1.5 mg/d), but responded marginally to five months of zinc (50 mg/d) supplementation. Simultaneous administration of L-carnosine, gradually increased from 0.5 g/d to 2 g/d over four months, markedly improved her symptoms. Five months after adding 2 g/d L-carnosine, the patient’s pronounced alternate identities that people around her could notice no longer appeared. However, the identities that were not noticeable to the people remained. They disappeared completely two years later and reappeared only when zinc and L-carnosine were discontinued during the subsequent three-year follow-up. The patient’s severity scores for dissociation and depression were reduced. Furthermore, signs of emotional/mood instability, flashbacks, binge eating, and self-harm improved. The physical symptoms of zinc deficiency eventually resolved. Further investigation of cotreatment with zinc and L-carnosine for DID and related conditions, particularly the contribution of zinc deficiency to dissociation, is necessary.

## Introduction

Dissociative identity disorder (DID) involves the disruption of a patient’s identity, characterized by two or more distinct personality states and recurrent gaps in the recall of everyday events [1]. However, little is known about the effectiveness of pharmacotherapy for DID [2]. Furthermore, no guidelines for pharmacotherapy of dissociative disorders currently exist [3]. Although the pathophysiology of dissociative disorders is poorly understood, glutamatergic hyperactivity has been suggested to occur in the brain [4].

Zinc is indispensable for proper physiological brain function. Its deficiency can induce cognitive impairment, mood lability, irritability, and impulsivity, which are commonly observed in individuals with dissociative symptoms [5,6]. Thus, zinc deficiency might be a contributor to dissociative disorders. L-carnosine , an endogenous dipeptide composed of β-alanine and L-histidine, is abundant in nerve tissue. It has various biological functions, including antioxidant, anti-inflammation, anti-glycation, pH buffering, metal ion chelating, and anti-aging activity, as well as a neuroprotective effect [7,8]. Both zinc and L-carnosine have anti-glutamatergic properties [5,6,8]. We previously described patients with dissociative symptoms who improved with a zinc-L-carnosine complex (polaprezinc), suggesting a role of zinc deficiency in dissociation and the potential benefits of dissociation treatment with a combination of zinc and L-carnosine [9]. Zinc acetate hydrate is prescribed in Japan for zinc supplementation in patients with hypozincemia. L-carnosine is available as a dietary supplement. Herein, we report the case of a patient with DID who had zinc deficiency and was successfully cotreated with 50 mg/d zinc (using zinc acetate hydrate) and 2 g/d L-carnosine.

## Case presentation

The patient was a 30-year-old unmarried Japanese woman with DID and bipolar I disorder who had experienced physical and verbal abuse by her strict mother since childhood as well as bullying from kindergarten to junior high school. Despite her desire to avoid attending school, her mother did not allow it. Until nine years of age, she imagined that a fairy lived in her school shoe closet, which was visible only to her, and acted as her friend. She would frequently talk to her reflection in the mirror in her room, sharing her daily annoyances. From the age of 11 years, she frequently experienced forgetfulness and made careless mistakes. She only had vague memories of her school days and was unable to recall the names of most of her classmates. She wanted to be emotionally detached and achieved this at age 15. Her father was indifferent and did not notice that her mother was abusive; he was also unaware of the bullying she experienced at school. Her mother suffered from depression and fibromyalgia. When the patient was 22, her parents divorced, and her mother left home.

The patient entered college at age 18. At age 21, she had a manic episode involving inflated self-esteem, hyperactivity, talkativeness, irritable mood, and extravagance, leading to interpersonal conflicts. This episode lasted for four months, and then, the patient experienced a depressive episode of one month. This was followed by a second manic episode of four months, a second depressive episode of one year, and then a residual depressive state. There were few periods of remission between these episodes. She did not receive any treatment during these episodes. At age 22, she graduated from college and attained a full-time job; however, she quit the job after two months. Soon afterward, during a depressive period, the patient was urgently admitted to a hospital for hyperventilation syndrome (dyspnea, dizziness, lightheadedness, palpitations, sweating, dry mouth, numbness, and stiffness), possibly with dissociative symptoms. During her 10-day hospital stay, she received risperidone (1 mg once daily), etizolam (0.5 mg three times daily), and supportive psychotherapy. After discharge, the patient attended a clinic and received mirtazapine (7.5 mg once daily), clonazepam (1 mg twice daily), ethyl loflazepate (1 mg once daily), and supportive psychotherapy for eight months but stopped attending by herself at age 23. At the time, her diagnosis was unknown.

By age 23, the people around her had noticed that she would abruptly switch to three alternate identities. The alternate identities had different characteristics from the primary identity. The first identity (A) was a woman with a strong and irritable personality who confronted those who caused stress to the primary identity and articulated thoughts that the primary identity was hesitant to express. Its speech and handwriting contrasted with those of the primary identity. The second identity (B) manifested as a continuously crying seven-year-old girl. The third identity (C) was a woman exhibiting a violent personality and homicidal ideation. The primary identity did not have any memory of the actions of the alternate identities. She later recalled the following: first, she experienced a fog in her head; she then felt being controlled by another person and hearing words in “red voices,” “blue voices,” or “cream voices;” and finally, her identities altered.

At age 24 (three months after the previous treatment), the patient visited our outpatient clinic during the third depressive episode. She presented with a depressed mood, energy loss, leaden paralysis, hypersomnia (15 hours of sleep per day), and dissociation (including identity alteration, occurring three to four times per week). The patient had a poor appetite but partook in occasional binge-eating periods lasting two to four weeks, during which she would binge eat one to three times per day, almost daily. She also exhibited emotional/mood instability as well as impulsivity (self-harm) and uncontrolled anger, evidenced by cutting her wrists, punching walls, and trashing her room. Furthermore, she experienced intrusive recollections of adverse experiences, flashbacks, and nightmares almost daily. Her flashbacks were related to the abuse from her mother and bullying from classmates. These flashbacks would last 15-30 minutes, during which she would panic, cover her ears, and wrap herself in a duvet. Her nightmares resembled a horror film: scary people chasing her or drowning her in a bathtub or all her teeth falling out. She experienced sleep paralysis during every nightmare. She did not use any medications, alcohol, or substances. Neurological examination revealed no significant findings. Blood tests for complete blood count; liver, renal, and thyroid functions; electrolytes; and diabetes revealed normal findings, with negative results for anti-Sjögren syndrome A/B and antinuclear antibodies tests ruling out autoimmune diseases. We diagnosed the patient with DID and bipolar I disorder, according to the Diagnostic and Statistical Manual of Mental Disorders, Fifth Edition [1]. A diagnosis of posttraumatic stress disorder (PTSD) was not made due to insufficient intensity of traumatic events.

Despite administration of aripiprazole (0.75 mg once daily) and clonazepam (0.5 mg three times daily), her symptoms did not improve over the next three months. Three months after the first visit, the Dissociative Experiences Scale (DES) and the 16-item Quick Inventory of Depressive Symptomatology (self-report; QIDS-SR_16_) were used to assess the severity of dissociation and depression, respectively [10,11]. She scored 46.1 on the DES and 24 on the QIDS-SR_16_. Generally, a DES score ≥30 indicates a possible dissociative disorder. A QIDS-SR_16_ score ≥11 indicates moderate to more severe depression. At this time, the patient also reported long-term physical symptoms of zinc deficiency, including hair loss, nail fragility, acne, dermatitis, dysgeusia, xerostomia, and dysphagia [12-14]. She therefore underwent a zinc taste test to assess the degree of zinc deficiency causing dysgeusia. Zinc sulfate solution was administered orally, and the patient’s response to the solution was graded on an ordinal scale of 1-4 [15]. She scored a 2, indicating moderate zinc deficiency. Her morning fasting serum levels were as follows: zinc, 85 μg/dL (reference range (RR), >80 μg/dL); copper, 85 μg/dL (RR, 70-132 μg/dL); iron, 79 μg/dL (RR, 40-180 μg/dL); and ferritin, 82.6 ng/mL (RR, 4.0-87.0 ng/mL) [12].

Although the patient was not hypozincemic, zinc deficiency was strongly suspected. Therefore, zinc acetate hydrate (Nobelzin, Nobelpharma Co., Ltd., Tokyo, Japan) containing 50 mg/d elemental zinc (25 mg twice daily) was added three months after the first visit, after she provided informed consent for off-label use. Aripiprazole and clonazepam were continued with the same dosages. After five months of zinc use, the patient’s emotional/mood instability and impulsivity improved slightly. However, dissociation, flashbacks, and fluctuations in appetite persisted with minimal changes. She scored 44.6 on DES and 19 on QIDS-SR_16_. Moreover, the physical symptoms of zinc deficiency did not improve significantly despite an elevated serum zinc level (zinc, 115 μg/dL; copper, 98 μg/dL; iron, 97 μg/dL; and ferritin, 46.5 ng/mL). Zinc acetate hydrate was then continued with the same dosage.

L-carnosine (NOW Foods, Bloomingdale, USA), a dietary supplement, was then added. The dosage was gradually increased over four months, from 0.5 g/d (0.5 g once daily) to 1 g/d (0.5 g twice daily), 1.5 g/d (0.5 g and 1 g once daily), and 2 g/d (1 g twice daily). Aripiprazole, clonazepam, and zinc acetate hydrate were continued with the same dosages. After one week of the L-carnosine (0.5 g/d) addition, binge eating decreased, and loss of appetite was alleviated, resulting in appetite stabilization. Within the next one month on L-carnosine (1 g/d), dissociation, flashbacks, nightmares, emotional/mood instability, and impulsivity improved. Increasing the dose of L-carnosine relieved symptoms that worsened under stress or fatigue. One month after administration of 2 g/d L-carnosine (five months after initiation of L-carnosine), the patient’s family reported a marked decrease in the frequency of alternate identities, from occurring every alternate day to only one to two times per month. Therefore, the patient was able to work again. After five months of treatment with 2 g/d L-carnosine, the alternate identities that people around her could notice no longer appeared. Subsequently, her alternate identities that were not noticeable to anyone appeared only when she was writing her diary; they appeared on three to four days of 10 to 15 days she wrote it per month. Two years later (41 months after the first visit), she received a farewell message from the alternate identity (A) that was written in her diary, “You don’t need me anymore, do you?” This implied that the personalities were integrated.

**Figure 1:**
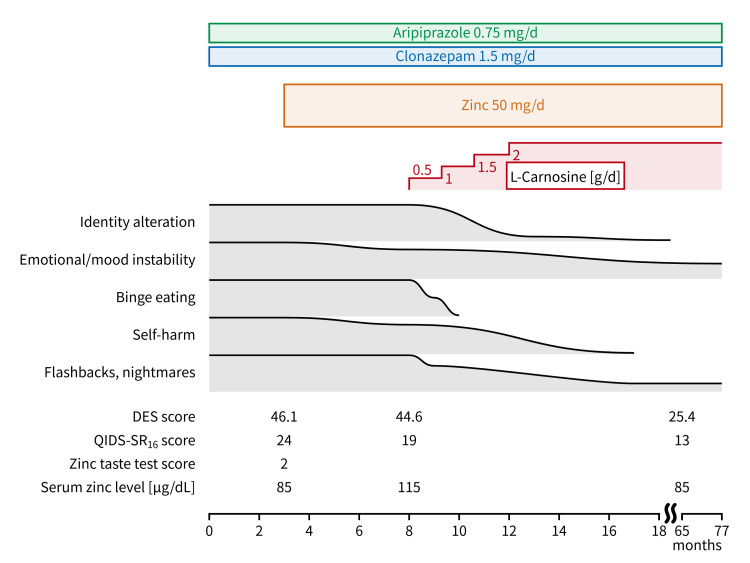
Course of treatment Bands indicate symptom trajectory. Between 18 and 46 months of treatment, the zinc dosage was 25 mg/d. At 41 months of treatment, the patient received a farewell message from alternate identity (A), and the identity alteration was resolved. However, it recurred when zinc and L-carnosine were discontinued several times thereafter (not shown here). DES: Dissociative Experiences Scale; QIDS-SR_16_: 16-item Quick Inventory of Depressive Symptomatology (self-report)

During the subsequent three-year follow-up (up to 77 months after the first visit), the alternate identities only emerged when zinc and L-carnosine were discontinued several times by herself while taking aripiprazole and clonazepam. Treatment discontinuation for two to four weeks led to recurrence. Approximately two years after receiving the farewell message (65 months after the first visit), her scores improved to 25.4 on DES and 13 on QIDS-SR_16_; the frequency of her flashbacks and nightmares decreased to two to three times per month and one to two times per week, respectively; and her physical symptoms were all resolved. Serum levels at this time were as follows: zinc, 85 μg/dL; copper, 90 μg/dL; iron, 66 μg/dL; and ferritin, 35.0 ng/mL. Between 18 and 46 months after the first visit, the zinc acetate hydrate dosage was reduced to 25 mg/d elemental zinc (25 mg once daily) because of nausea, and then increased back to 50 mg/d elemental zinc (25 mg twice daily) at the patient’s request. Throughout the treatment period, no other adverse effects were observed clinically or in routine blood test results while the patient was taking all the medications and L-carnosine at the above dosages. Furthermore, the patient had had no manic episodes since the first visit. The changes in the serum levels of biochemical variables are summarized in Table 1. The treatment course is depicted in Figure 1.

**Table 1:** Changes in biochemical variables over the course of treatment (morning fasting serum levels)

Variable	Before zinc treatment	After five months of zinc treatment and before L-carnosine treatment	After recovery
Zinc (μg/dL)	85	115	85
Copper (μg/dL)	85	98	90
Iron (μg/dL)	79	97	66
Ferritin (ng/mL)	82.6	46.5	35.0

The author provided only minimal supportive psychotherapy throughout the treatment period in our clinic. This was a session of approximately 10 minutes each, offered as part of a routine consultation when the patient visited every two weeks, until 32 months after the first visit, switching to every four weeks thereafter. The session focused on supporting her through listening, acceptance, and empathy.

The patient reported that she was emotionally stable with zinc and L-carnosine, and that the only side effect from these agents during the treatment period was nausea, which was tolerable after the zinc was increased again to the original dosage. Furthermore, she stated that her intrusive recollections and flashbacks had become less frequent, so she no longer panicked unless she intentionally recalled unpleasant memories from the past. She was able to return to work full-time.

After follow-up, aripiprazole and clonazepam dosages are currently being tapered at the patient’s request, and zinc and L-carnosine are ongoing at the same dosages. She is not receiving any therapy, other than supportive psychotherapy.

## Discussion

To the best of our knowledge, this is the first report of a patient with DID who was successfully cotreated with zinc and L-carnosine. The patient exhibited varied mental symptoms that responded minimally to five months of zinc supplementation, but she showed marked improvement upon the introduction of L-carnosine. In particular, the improvement in her dissociative symptoms was noteworthy, as shown by a decrease in her DES score to below the cut-off value for dissociative disorders. The patient developed dissociation in childhood, which manifested as DID in adulthood. We hypothesized that zinc deficiency may contribute to dissociation because a lack of zinc can induce mental symptoms, which mostly correspond with those associated with dissociation. Two rationales indicated that our patient with DID had a zinc deficiency. First, she exhibited several physical symptoms of zinc deficiency, which were eventually resolved with zinc supplementation. A diagnosis of zinc deficiency should rely on the treatment response to zinc supplementation because serum zinc levels do not necessarily reflect cellular zinc status [16,17]. Second, the result of the pre-treatment zinc taste test indicated a zinc deficiency. In addition, her dissociative symptoms improved in parallel with her physical symptoms of zinc deficiency. Five months of zinc monotherapy was insufficient to resolve these symptoms; however, a prolonged period of combined zinc supplementation with L-carnosine administration was effective. This may be due to the zinc ionophore activity of L-carnosine, which promotes the intracellular uptake of zinc [18]. Thus, if dissociation is due to zinc deficiency, combining L-carnosine with zinc may be a more advantageous treatment strategy than using zinc alone.

Dissociation is related to childhood trauma, and DID is strongly associated with PTSD [19]. Although our patient did not fully satisfy the criteria for PTSD, she had PTSD-like symptoms, including intrusive recollections, flashbacks, and nightmares, that are closely linked to dissociation. Zinc and L-carnosine have two common beneficial roles in the treatment of dissociation and PTSD. First, both have anti-glutamatergic effects against dissociation. Zinc binds to and inhibits the glutamate N-methyl-D-aspartate receptor [5,6]. L-carnosine enhances cellular glutamate reuptake by upregulating the glutamate transporter GLT1 [8]. Second, both have antioxidant and anti-inflammatory properties that inhibit the oxidative stress and neuroinflammation that causes PTSD [8,20,21]. L-carnosine has two additional benefits against PTSD. Elevated L-carnosine levels in the brain reduce PTSD-like behaviors by maintaining brain-derived neurotrophic factor expression in the hippocampus [22]. Patients with PTSD show a decreased threshold for amygdala activation to trauma-related stimuli [23]. However, L-carnosine ameliorates amygdaloid-kindled seizures via the histaminergic action derived from its component, L-histidine [24].

Our patient had comorbid bipolar disorder with binge eating and impulsivity (self-harm) that benefited from zinc and L-carnosine. The pathophysiology of mood disorders includes glutamatergic hyperactivity, oxidative stress, and neuroinflammation [25,26]. These can be reduced using zinc and L-carnosine. These agents have been shown to have anti-depressant effects in depressed patients [27,28]. In our patient, the change in the QIDS-SR_16_ score indicated a reduction in her depression level from very severe to moderate.

Binge eating and self-harm exhibited almost complete remission. Binge eating and impulsivity are common in individuals with dissociation [29,30]. Moreover, binge eating is considered an impulsive behavior and is associated with glutamatergic hyperactivity [31]. We previously described the effectiveness of polaprezinc (consisting of 34 mg/d zinc and 116 mg/d L-carnosine) in treating binge eating [9,14]. The patient’s binge eating symptoms responded more rapidly and clearly to higher doses of zinc and L-carnosine in the present study than in previous studies. Zinc deficiency is possibly involved in binge eating. Zinc and L-carnosine help against binge eating, possibly through their anti-glutamatergic properties and the histaminergic properties of L-carnosine [14]. Impulsivity has been reported to be positively correlated with glutamate levels and negatively correlated with γ-aminobutyric acid (GABA) levels in the brain [32]. L-carnosine has anti-glutamatergic and GABA-enhancing properties, suggesting that it might be beneficial in treating impulsivity [33].

Our patient possibly had attention-deficit/hyperactivity disorder (ADHD) because she exhibited symptoms of inattention even after improvement in dissociation. Nevertheless, a diagnosis of ADHD was difficult because of overlapping severe dissociations since childhood. ADHD is related to increased glutamate levels in the brain [34]. Hence, ADHD, if present, may have adversely affected and partly contributed to our patient’s symptoms.

As this is a single case study, we cannot exclude the possible effects of a placebo or time-course response. In addition, DES, QIDS-SR_16_, and the zinc taste test were not conducted at the first visit. Nevertheless, the inhibition of identity alteration for more than five years and the temporal relationship between several episodes of treatment discontinuations and recurrence support the effectiveness of this treatment regimen. Furthermore, as shown in our patient, zinc and L-carnosine have few significant adverse effects and no risk of overdose.

## Conclusions

Cotreatment with zinc and L-carnosine proved to be effective for treating our patient with DID who had zinc deficiency. DID and related symptoms, including PTSD-like symptoms, emotional/mood instability, binge eating, and impulsivity, have a potential shared underlying mechanism of glutamatergic hyperactivity. This can be treated with zinc and L-carnosine. There are currently few effective medications for DID. Therefore, this treatment regimen for DID and related conditions, along with the role of zinc deficiency in dissociation, merits further exploration.
